# Consensus guidelines on the bedside assistant skills required in robotic surgery

**DOI:** 10.1007/s00464-024-11206-x

**Published:** 2024-09-03

**Authors:** Riley Brian, Alyssa Murillo, Camilla Gomes, Hueylan Chern, Daniel Oh, Patricia S. O’Sullivan

**Affiliations:** 1 Department of Surgery, University of California San Francisco, 513 Parnassus Avenue, S-321, San Francisco, CA 94143 USA; 2 Department of Surgery, University of Southern California, Los Angeles, CA USA; 3 Intuitive Surgical, Sunnyvale, CA USA

**Keywords:** Robotic surgery, Bedside assistance, Technical skills, Non-technical skills

## Abstract

**Background:**

While bedside assistants play a critical role in many robotic operations, substantial heterogeneity remains in bedside assistant training pathways. As such, this study aimed to develop consensus guidelines for bedside assistant skills required for team members in robotic operations.

**Methods:**

We designed a study using the Delphi process to develop consensus guidelines around bedside assistant skills. We generated an initial list of bedside assistant skills from the literature, training materials, and expert input. We selected experts for the Delphi process based on prior scholarship in the area of robotic bedside assistant education and experience facilitating robotic bedside assistant training. For each item, respondents specified which robotic team members should have the skill from a list of “basic” bedside assistants, “advanced” bedside assistants, surgeons, surgical technologists, and circulating nurses. We conducted two rounds of the Delphi process and defined 80% agreement as sufficient for consensus.

**Results:**

Fourteen experts participated in two rounds of the Delphi process. By the end of the second round, the group had reached consensus on 253 of 305 items (83%). The group determined that “basic” bedside assistants should have 52 skills and that “advanced” bedside assistants should have 60 skills. The group also determined that surgeons should have 54 skills, surgical technologists should have 25 skills, and circulating nurses should have 17 skills. Experts agreed that all participants should have certain communication skills and basic knowledge of aspects of the robotic system.

**Conclusions:**

We developed consensus guidelines on the skills required during robotic surgery by bedside assistants and other team members using the Delphi process. These findings can be used to design training around bedside assistant skills and assess team members to ensure that each team member has the appropriate skills. Hospitals can also use these guidelines to standardize expectations for robotic team members.

**Supplementary Information:**

The online version contains supplementary material available at 10.1007/s00464-024-11206-x.

Bedside assistants play a critical role in many robotic operations [1]. By managing the robotic system and providing adjunct laparoscopic support, bedside assistants can facilitate case progression and patient safety [2]. Multiple studies have suggested that the experience of the bedside assistant affects important outcomes in robotic surgery [3–6]. Thus, education and training pathways for bedside assistants must adequately prepare them to be effective in the operating room [7].

Despite the importance of appropriate instruction for bedside assistants, existing training programs, case requirements, and credentialing processes vary substantially [7–10]. Many authors report curricula requiring learners to participate in as few as five cases before being considered competent; however, prior learning curve analyses suggest that bedside skills continue to mature over dozens of cases [1]. Structured education in robotic surgery has tended to de-emphasize bedside assistant skills in favor of a greater focus on the training of the console surgeon [11, 12].

Importantly, bedside assistants come from a wide variety of educational backgrounds—including board-certified surgeons, physician assistants, nurses, surgical technologists, and residents [1]. More recently, medical students have trained to work as bedside assistants [13–15]. In the setting of such diverse expertise, several authors have identified key components of bedside assistant training [16, 17]. Building on these suggestions, both the Association of Surgical Technologists and the Association of Surgical Assistants have published guidelines outlining necessary skills for those working at the robotic bedside [18, 19]. Together, these publications indicate skills needed for bedside assistants in the pre-operative, intra-operative, and post-operative settings [16–19]. Nonetheless, skills described in prior publications vary in scope, highlight different skills, and arose from authors’ perceptions.

Prior work has specifically called for standardization of robotic surgical curricula both for education and credentialing purposes [20]. By better defining the skills needed of bedside assistants, we can clarify training pathways and ground credentialing decisions in evidence. As such, this study aimed to develop consensus guidelines from diverse experts for the bedside assistant skills required in robotic operations by specific roles and across educational backgrounds. We hypothesized that experts would identify required skills for bedside assistants and other robotic surgery team members that vary based on role.

## Methods

### Study design

**Fig. 1:**
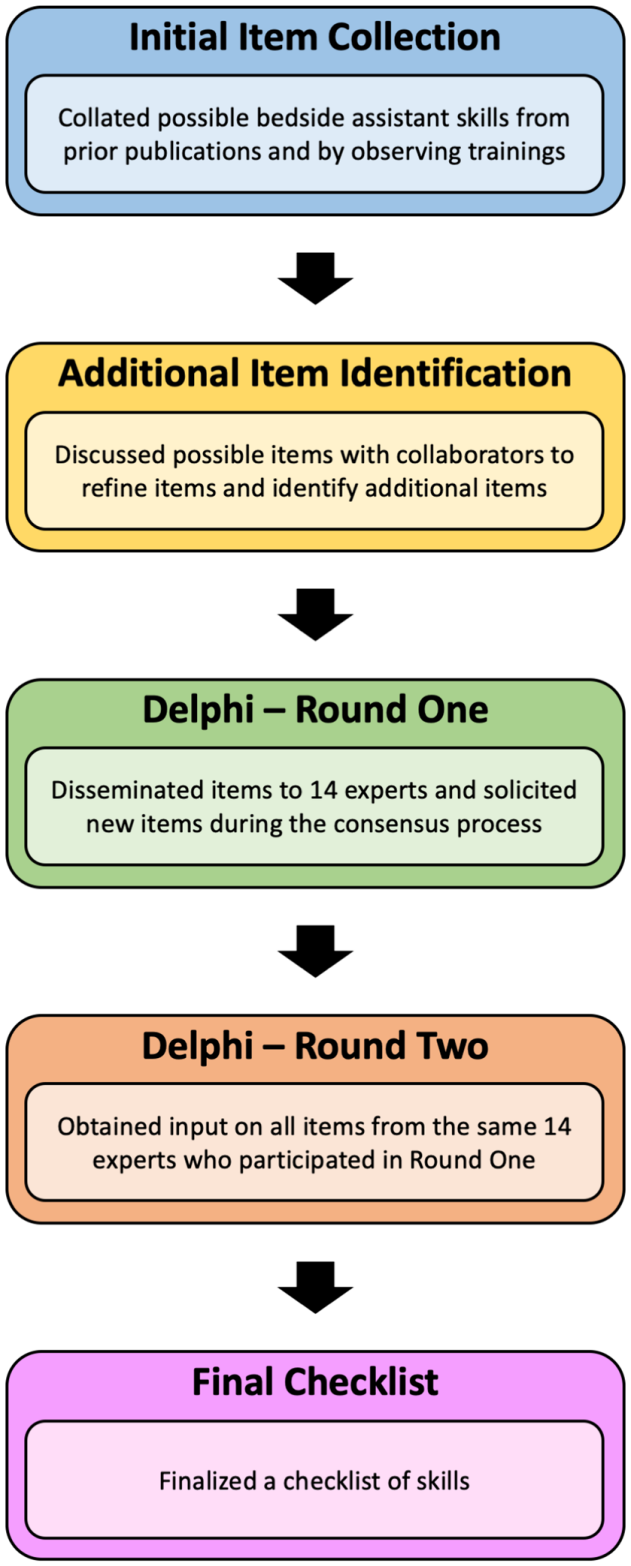
Evolution of items during the study

We developed consensus guidelines for the bedside assistant skills required for robotic operations using the Delphi process (Fig. 1) [21]. We chose to use a consensus group method of guideline generation given the lack of trial-based data to support a standardized list, and we selected the Delphi process for reasons of cost and due to the geographic separation of experts [22].

### Item development

We developed an initial list of bedside assistant skills based on prior peer-reviewed and society-based publications suggesting skills useful for bedside assistants [16–19]. We collated all previously published items and adapted them if needed to create consistency of phrasing and eliminate redundancies. Multiple authors attended bedside assistant training courses run by the University of California San Francisco (UCSF) and Intuitive Surgical and discussed the candidate items with external collaborators (see acknowledgements) to identify additional items (Intuitive Surgical, Sunnyvale, CA). For each item, we asked respondents to specify which robotic team members should have the skill from a list of “basic” bedside assistants, “advanced” bedside assistants, surgeons, surgical technologists, and circulating nurses. We defined “basic” bedside assistants as those working in cases without an assist port and “advanced” bedside assistants as those working in cases with an assist port. We chose to identify skills for non-bedside assistant team members (i.e., surgeons, surgical technologists, and circulating nurses) to allow for comparisons in skill requirements among roles. We used a scale from one to four for each item, which ranged from “definitely does not need the skill” for one to “definitely needs the skill” for four. Four authors piloted the questionnaire and we made additional modifications prior to finalizing the items (available as Supplementary File 1).

### Expert identification and recruitment

We selected experts based on two major criteria: prior scholarship in the area of robotic bedside assistant education and prior experience conducting robotic bedside assistant training. We identified experts meeting the first criterion through a literature review, and experts meeting the second criterion through discussion with the Intuitive Surgical professional education team. We aimed to recruit a diverse group of experts representing bedside assistants and surgeons from multiple surgical specialties.

After generating an initial list of experts, we individually emailed each candidate and explained the reason for selection and the study’s design and purpose. We offered experts a gift card incentive for each round in which they participated.

### Delphi process

After candidates expressed an interest in participating, we individually disseminated the first round of items with an email containing a Qualtrics link (Qualtrics, Provo, UT). We did not ask participants for the rationale behind their responses. After experts participated in the first round, we collated responses to identify the mean score assigned for each item. Prior to starting the study, we determined that 80% consensus would be required for an item to be included as a skill. We defined 80% consensus as having 80% of respondents state that a team member “probably” or “definitely” needs the skill described by the item based on the four-point scale (Supplementary File 1). We calculated Cronbach’s alpha in each round using StataNow/BE 18.5 for Mac (StataCorp, College Station, TX). We maintained total anonymity among participants throughout the process. Participants did not meet at any time.

We included those items with less than 80% consensus after the first round in the second round. We also incorporated new items based on experts’ suggestions. We then asked the same experts to participate in a second round. If an item did not achieve 80% consensus by the end of the second round, we deigned to not include them item in the final list due to lack of expert clarity about the necessity of the item. We disseminated final results to all participants at the end of the second round.

### Ethical considerations

Our Institutional Review Board determined this study to be exempt from review (IRB23-39,876).

## Results

We recruited 14 experts to participate in the two rounds of the Delphi process. All 14 experts (100%) who expressed interest in participating before starting the process subsequently completed both rounds. Experts included six surgeons, four physician assistants, two certified surgical assistants, one nurse, and one surgical technologist who also worked as a robotic coordinator. Thirteen experts practiced in eight geographically diverse states in the United States while one expert practiced internationally. Experts represented specialties within general surgery, obstetrics-gynecology, and urology. Experts had participated in a median of 1650 robotic cases (IQR 1000-3500).

The review of items resulted in 59 items for consideration across five different team members, resulting in 295 decisions for each expert. All 14 experts completed all 295 selections. In the first round, the group reached consensus on 220 of 295 decisions (75%) with Cronbach’s alpha of 0.98. More specifically, the group reached consensus regarding 47 of 59 items (80%) for “basic” bedside assistant skills and 55 of 59 items (93%) for “advanced” bedside assistant skills. They also reached consensus on 53 of 59 items (89%) for surgeon skills, 21 of 59 items (36%) for surgical technologist skills, and 44 of 59 items (75%) for circulating nurse skills. Experts added two unique skills—related to manually releasing instruments and loading/unloading staplers—to the 59 original skills during the first round of the Delphi process resulting in a list of 61 skills.

In the second round, all 14 experts made 84 of the 85 selections (98.8%). One expert did not respond to one of the items, though the item reached consensus and this would not have changed based on the fourteenth expert’s response. The group reached consensus on 33 of 85 unique items (39%) with Cronbach’s alpha of 0.95. The group reached consensus on 5 of 14 items (36%) for “basic” bedside assistant skills and 5 of 6 items (83%) for “advanced” bedside assistant skills. They also reached consensus on 2 of 8 items (25%) for surgeon skills, 15 of 40 items (38%) for surgical technologist skills, and 6 of 17 items (35%) for circulating nurse skills.

Thus, by the end of the second round, the group had reached consensus on 253 of the 305 unique items (83%). The group determined that “basic” bedside assistants should have 52 skills and that “advanced” bedside assistants should have 60 skills. They also determined that surgeons should have 54 skills, surgical technologists should have 25 skills, and circulating nurses should have 17 skills. Skills spanned pre- and post-operative technical skills (Table 1), intra-operative technical skills (Table 2), and non-technical skills (Table 3). There was substantial overlap between skill requirements for bedside assistants and surgeons, with less overlap in skill requirements among bedside assistants, surgical technologists, and circulating nurses.

**Table 1:**
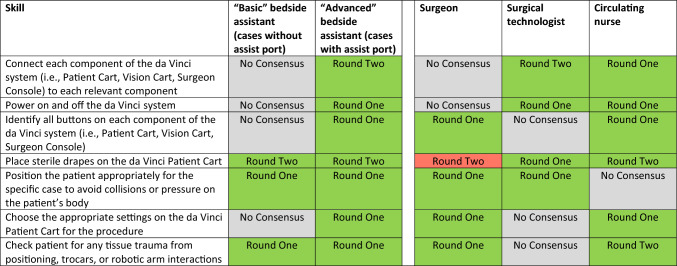
Pre- and post-operative technical skill list (Color table online)

In round one and round two of the Delphi process, experts came to consensus on pre- and post-operative technical skills that bedside assistants and other team members should have (green) or do not need (red) in a robotic operation. Experts did not come to consensus on the necessity of team members having a number of the skills (gray)

**Table 2:**
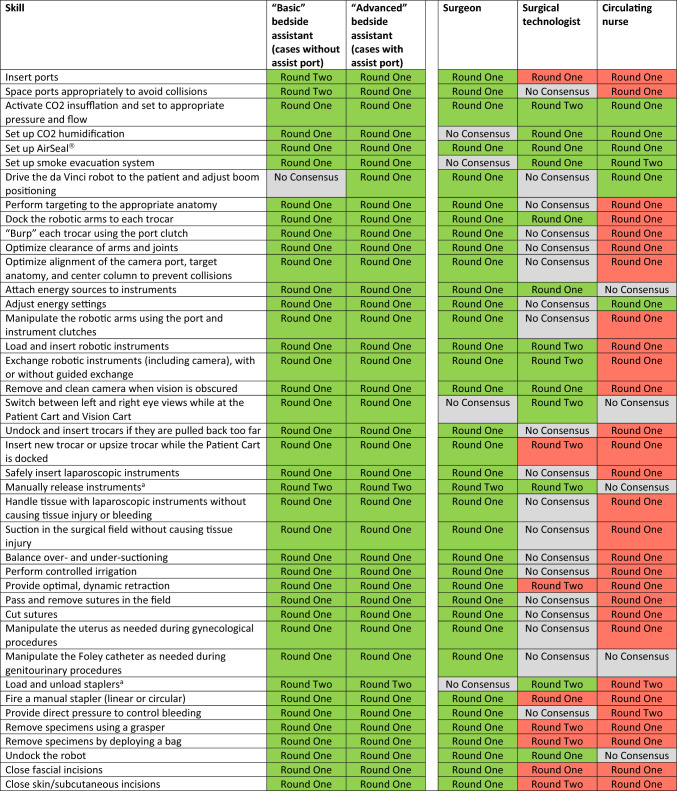
Intra-operative technical skill list (Color table online)

In round one and round two of the Delphi process, experts came to consensus on intra-operative technical skills that bedside assistants and other team members should have (green) or do not need (red) in a robotic operation. Experts did not come to consensus on the necessity of team members having a number of the skills (gray)

^a^Added in Round Two

**Table 3:**
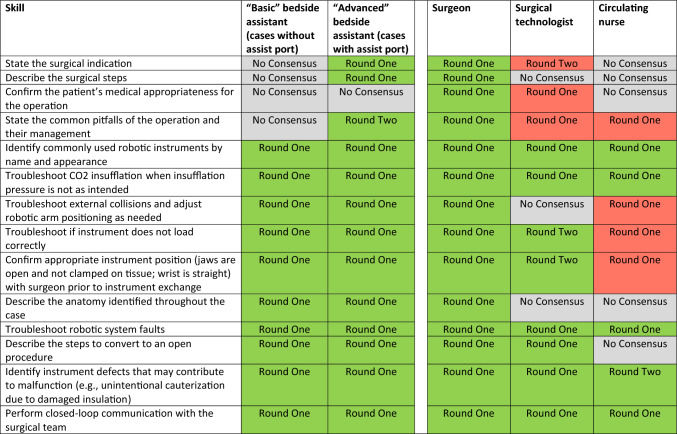
Non-technical skill list (Color table online)

In round one and round two of the Delphi process, experts came to consensus on non-technical skills that bedside assistants and other team members should have (green) or do not need (red) in a robotic operation. Experts did not come to consensus on the necessity of team members having a number of the skills (gray)

## Discussion

In this study, we used the Delphi process to develop consensus guidelines on the bedside assistant skills required during robotic surgery. We identified 61 skills related to robotic bedside assistance and experts determined whether bedside assistants and other team members should have each skill. Experts deemed all 61 skills to be required of at least one team member in an operation, though there was no agreement as to which team members needed some of the skills. Overall, these findings can be used to design training around bedside assistant skills and assess team members to ensure that each team member has the appropriate skills. We anticipate that hospitals can use these guidelines—rather than team members’ degrees—to standardize expectations for robotic team members around bedside assistant skills.

We noted several interesting findings with regard to consensus items. Experts agreed that “basic” bedside assistants and “advanced” bedside assistants should have most of the intra-operative technical skills included in the list as candidate items. We speculate that all intra-operative technical skills may be viewed as essential given the possibility of unexpected events during the course of any operation. Experts most strongly agreed on the importance of communication in robotic operations. Given the unique physical set up of the robotic operating room, this finding supports prior work highlighting the centrality of communication to the success of robotic surgery [23, 24]. Experts disagreed more substantially around whether bedside assistants needed certain pre- and post-operative technical skills and non-technical skills related to knowledge around procedures and patients. Finally, we found quite varied opinions as to the skills required of surgical technologists. This likely reflects the diverse practice settings of the experts included in this study, as well as more general variability in surgical technologists’ role in different procedures. We had chosen to include surgeons, surgical technologists, and circulating nurses as comparator groups to contrast their necessary skills with those of bedside assistants.

This study builds on prior work establishing the necessary skills for bedside assistants and other team members in robotic surgery. One prior consensus study identified possible credentialing requirements in robotic surgery, though limited proposals to operating surgeons [12]. Other guidelines have focused on bedside assistant skills, though these stemmed from authors’ perceptions rather than expert consensus [16–19]. These guidelines formed the basis for many of the items we evaluated in this study. Our work adds to these prior publications by its consensus-based approach to defining necessary skills for bedside assistants. Of note, we did not ask experts to stratify skills for bedside assistants beyond “basic” and “advanced” cases. Certainly, those in different roles (e.g., physician assistant vs surgical resident) and with different experience levels may be expected to demonstrate “basic” or “advanced” skills at various times in their career or training depending on institution-specific case volumes and policies. Institutions may use the guidelines developed here to determine when bedside assistants are prepared to participate in different cases. Such decisions can stem from skill rather than degree or prior time spent assisting.

There are multiple limitations within our study that moderate our findings. First, our developed list contains many items specific to the da Vinci surgical system. As emerging robotic surgical systems become available, additional work must generalize this list to reduce its platform specificity. Second, our experts included a limited number of surgical technologists and nurses as the study’s focus was on the skills required of bedside assistants. As such, experts may not have had the insight to appropriately determine the skills needed for each role. We defined expertise based on publications and experience conducting bedside assistant training, though other methods of expert selection may have led to different results. Additionally, 13 of our 14 experts practiced in the United States, which may limit the generalizability of these findings internationally. Similarly, the inclusion of an international expert, despite that expert’s prior experience publishing about bedside assistant training, may limit results given global practice pattern variation. Furthermore, there are also limitations to the application of our findings. Experts did not come to consensus on a portion of the items. We opted to end the process after the second round as we perceived the lack of consensus at this point to reflect the quite varied practice patterns around the country and world. For example, experts seemed to identify quite disparate roles for surgical technologists. These different practice patterns may challenge the use of the list developed here. Future work could aim to further standardize role-specific expectations; nonetheless, focusing on standardizing the skills required of all bedside assistants may have broad applicability.

## Conclusion

We used the Delphi process to develop consensus guidelines around the skills required for bedside assistants in robotic surgery. These guidelines may serve as a framework for those designing training and assessments for bedside assistants. Standardized expectations and requirements for bedside assistant skills may allow for the consistency and quality needed in robotic surgery.

## Supplementary Information

Below is the link to the electronic supplementary material.

Supplementary file1 (DOCX 28 KB)
