# Supplementary material for: Consensus guidelines on the bedside assistant skills required in robotic surgery
Source: Surg Endosc. 2024 Sep 3;38(11):6406–12. doi: 10.1007/s00464-024-11206-x (PMC11525406; doi:10.1007/s00464-024-11206-x)
Supplement: Supplementary file 1 — Supplementary file1 (DOCX 28 KB) [file 464_2024_11206_MOESM1_ESM.docx]

Supplementary File 1: Questionnaire

For each skill in the left-hand column, rate whether each operating room team member should have the skill on a scale from 1 to 4:

1 = "definitely does not need the skill"
2 = "probably does not need the skill"
3 = "probably needs the skill"
4 = "definitely needs the skill"

|  | Surgeon (1) | “Advanced” bedside assist (cases with assist port) (2) | “Basic” bedside assist (cases without assist port) (3) | Surgical technologist (4) | Circulating nurse (5) |
| --- | --- | --- | --- | --- | --- |
| Connect each component of the da Vinci system (i.e., Patient Cart, Vision Cart, Surgeon Console) to each relevant component (1) |  |  |  |  |  |
| Power on and off the da Vinci system (2) |  |  |  |  |  |
| Identify all buttons on each component of the da Vinci system (i.e., Patient Cart, Vision Cart, Surgeon Console) (3) |  |  |  |  |  |
| Place sterile drapes on the da Vinci Patient Cart (4) |  |  |  |  |  |
| State the surgical indication (5) |  |  |  |  |  |
| Describe the surgical steps (6) |  |  |  |  |  |
| Confirm the patient’s medical appropriateness for the operation (7) |  |  |  |  |  |
| State the common pitfalls of the operation and their management (8) |  |  |  |  |  |
| Position the patient appropriately for the specific case to avoid collisions or pressure on the patient’s body (9) |  |  |  |  |  |
| Insert ports (10) |  |  |  |  |  |
| Space ports appropriately to avoid collisions (11) |  |  |  |  |  |
| Activate CO2 insufflation and set to appropriate pressure and flow (12) |  |  |  |  |  |
| Set up CO2 humidification (13) |  |  |  |  |  |
| Set up AirSeal (14) |  |  |  |  |  |
| Troubleshoot CO2 insufflation when insufflation pressure is not as intended (15) |  |  |  |  |  |
| Set up smoke evacuation system (16) |  |  |  |  |  |
| Choose the appropriate settings on the da Vinci Patient Cart for the procedure (17) |  |  |  |  |  |
| Drive the da Vinci robot to the patient and adjust boom positioning (18) |  |  |  |  |  |
| Perform targeting to the appropriate anatomy (19) |  |  |  |  |  |
| Dock the robotic arms to each trocar (20) |  |  |  |  |  |
| “Burp” each trocar using the port clutch (21) |  |  |  |  |  |
| Optimize clearance of arms and joints (22) |  |  |  |  |  |
| Optimize alignment of the camera port, target anatomy, and center column to prevent collisions (23) |  |  |  |  |  |
| Troubleshoot external collisions and adjust robotic arm positioning as needed (24) |  |  |  |  |  |
| Identify commonly used robotic instruments by name and appearance (25) |  |  |  |  |  |
| Attach energy sources to instruments (26) |  |  |  |  |  |
| Adjust energy settings (27) |  |  |  |  |  |
| Manipulate the robotic arms using the port and instrument clutches (28) |  |  |  |  |  |
| Load and insert robotic instruments (29) |  |  |  |  |  |
| Troubleshoot if instrument does not load correctly (30) |  |  |  |  |  |
| Confirm appropriate instrument position (jaws are open and not clamped on tissue; wrist is straight) with surgeon prior to instrument exchange (31) |  |  |  |  |  |
| Exchange robotic instruments (including camera), with or without guided exchange (32) |  |  |  |  |  |
| Remove and clean camera when vision is obscured (33) |  |  |  |  |  |
| Switch between left and right eye views while at the Patient Cart and Vision Cart (34) |  |  |  |  |  |
| Describe the anatomy identified throughout the case (35) |  |  |  |  |  |
| Undock and insert trocars if they are pulled back too far (36) |  |  |  |  |  |
| Insert new trocar or upsize trocar while the Patient Cart is docked (37) |  |  |  |  |  |
| Safely insert laparoscopic instruments (38) |  |  |  |  |  |
| Handle tissue with laparoscopic instruments without causing tissue injury or bleeding (39) |  |  |  |  |  |
| Suction in the surgical field without causing tissue injury (40) |  |  |  |  |  |
| Balance over- and under-suctioning (41) |  |  |  |  |  |
| Perform controlled irrigation (42) |  |  |  |  |  |
| Provide optimal, dynamic retraction (43) |  |  |  |  |  |
| Pass and remove sutures in the field (44) |  |  |  |  |  |
| Cut sutures (45) |  |  |  |  |  |
| Manipulate the uterus as needed during gynecological procedures (46) |  |  |  |  |  |
| Manipulate the Foley catheter as needed during genitourinary procedures (47) |  |  |  |  |  |
| Fire a manual stapler (linear or circular) (48) |  |  |  |  |  |
| Provide direct pressure to control bleeding (49) |  |  |  |  |  |
| Remove specimens using a grasper (50) |  |  |  |  |  |
| Remove specimens by deploying a bag (51) |  |  |  |  |  |
| Undock the robot (52) |  |  |  |  |  |
| Close fascial incisions (53) |  |  |  |  |  |
| Close skin/subcutaneous incisions (54) |  |  |  |  |  |
| Check patient for any tissue trauma from positioning, trocars, or robotic arm interactions (55) |  |  |  |  |  |
| Troubleshoot robotic system faults (56) |  |  |  |  |  |
| Describe the steps to convert to an open procedure (57) |  |  |  |  |  |
| Identify instrument defects that may contribute to malfunction (e.g., unintentional cauterization due to damaged insulation) (58) |  |  |  |  |  |
| Perform closed-loop communication with the surgical team (59) |  |  |  |  |  |

Q6 Are there any skills that you would like to reword?

________________________________________________________________

Q7 Are there any skills missing here that you would like to add?

________________________________________________________________

| Page Break |  |
| --- | --- |

What is your name?

________________________________________________________________

In about how many robotic cases have you participated?

________________________________________________________________

End of Block: Default Question Block
